# Mad Stone

**Published:** 1870-10

**Authors:** 


### MAD STONE.

John Smith, of New Hermon, Indiana, claimed several years ago that he had a mad stone, which will cure a bee-sting in a minute. He said the Indians with whom he had lived, claimed to get it from the rennett of a deer. Webster says, the rennett, or runnett, is the concreted milk found in the stomach of a sucking quadruped ; this may possibly suck up the poison as a sponge would suck up water-drops on a table, or it may contain a chemical substance which would antagonize the poison.

